# A Case of Spontaneous Coronary Artery Dissection in a Patient with Severe Anorexia Nervosa

**DOI:** 10.1155/2021/5526022

**Published:** 2021-06-23

**Authors:** Jessica O'Neil, Soumyaa Mazumder, Danita Yoerger Sanborn, Samantha Wu

**Affiliations:** ^1^Department of Medicine, Massachusetts General Hospital, Massachusetts, USA; ^2^Harvard Medical School, Massachusetts, USA; ^3^Department of Cardiology, Massachusetts General Hospital, Massachusetts, USA; ^4^Hospital Medicine Unit Massachusetts General Hospital, Massachusetts, USA

## Abstract

There are a variety of cardiac complications of anorexia nervosa including arrythmias, valvopathies, and myopathies. Spontaneous coronary artery dissection (SCAD) has not been widely reported among this patient population. This case report describes a middle-aged female with severe anorexia nervosa, who presented after being found unconscious, and was later diagnosed with SCAD. A literature review revealed one previous case of SCAD in a patient with anorexia nervosa and prompted a discussion of a series of possible predisposing factors for SCAD in this patient population. Patients with anorexia nervosa may be at increased risk for SCAD due to their complex nutritional and endocrine imbalances. This case highlights a possible underdiagnosed cardiac complication of anorexia nervosa.

## 1. Introduction

SCAD is a significant cause of acute coronary syndrome (ACS) in young and middle-aged women, accounting for nearly a quarter of ACS in women under the age of 50 [1]. Although advances in intracoronary imaging have helped increase the recognition of SCAD, it continues to be underdiagnosed [2]. SCAD has primarily been associated with predisposing arteriopathies (e.g., fibromuscular dysplasia and connective tissue disorders), high-stress conditions (e.g., intense exercise and significant emotional stress), and hormonal triggers (e.g., pregnancy). There has been little reported about the connection between SCAD and AN. AN is an illness marked by several nutritional and endocrine imbalances. It is associated with a number of cardiovascular complications including arrhythmias, heart failure, valvular disease, and sudden cardiac death [3]. This case raises the question of whether SCAD is an underappreciated diagnosis amongst patients with AN and may contribute to the high rate of cardiac morbidities in this population.

## 2. Case Presentation

A 55-year-old woman with a severe anorexia nervosa (AN) was found unresponsive for an unknown period of time with hypoglycemia (POCT glucose 14 mg/dL) and hypothermia (90.2°F). Her mentation and temperature normalized with intravenous dextrose and rewarming. She did not recall the events leading up to her presentation. She denied suicidal ideation or intentional ingestion. She reported previous mild dyspnea on exertion but denied chest pain. A few weeks prior to her presentation her father passed away leading to significant emotional stress. She was diagnosed with AN at age 18 which was primarily restrictive subtype; however, she did report prior episodes of purging. Her medical history also included hypothyroidism, for which she was on 125 mcg levothyroxine daily, hyponatremia, and iron deficiency anemia. On examination, she weighed 35 kg with a body mass index (BMI) of 12. Cardiac exam revealed regular rate and rhythm, normal S1 and S2, no murmurs, and no jugular venous distension. There was pitting edema to her ankles bilaterally. She was also noted to have gingival swelling and a petechial rash over her lower extremities, raising clinical concern for scurvy.

Laboratory values revealed hypomagnesemia (1.5 mmol/L), hypocalcemia (6.5 mgl/dL), and hypoalbuminemia (2.7 g/dL). Serum creatinine was 0.84 mg/dL. Thyroid-stimulating hormone (TSH) was within normal limits (1.27 uIU/mL) and triiodothyronine (T3) was at the lower bound of the normal range (76 ng/dL) though free thyroxine (FT4) was elevated to 3.3 ng/dL. This was attributed to a supratherapeutic dose of levothyroxine. Vitamin C (0.3 mg/dL) and 25–OH vitamin D (21.9 ng/mL) levels were low. Investigation also demonstrated an elevation in high sensitivity troponin elevated to 129 ng/L peaking to 364 ng/dL. ECG revealed Q-waves and ST elevation in leads V1-V3 c/w a septal infarct, new compared to an ECG from 4 months prior. Transthoracic echocardiogram (TTE) revealed an ejection fraction of 52% with akinesis of the septum at the midventricular level and hypokinesis of the apical septum. There was moderate mitral regurgitation. These abnormalities were new compared to a TTE from 2 years prior. A computed tomography coronary angiogram showed no atherosclerosis but demonstrated a linear hypodensity within the proximal segment of the first septal perforator artery and abrupt tapering of the vessel 5 mm from the origin consistent with SCAD. Cardiology was consulted and recommended conservative therapy with initiation of aspirin, metoprolol, and cardiac rehabilitation.

## 3. Discussion

SCAD is caused by the formation of an intramural hematoma (IMH) within the coronary artery wall that compresses the true lumen leading to ischemia. There are two leading theories describing of how the IMH arises: (1) it develops from damage to the vascular endothelium (possibly from an intimal tear), allowing blood to pool in a false lumen, or (2) the IMH forms following a spontaneous rupture of the vasa vasorum within the arterial wall [4]. There has been one previously reported case of SCAD in a patient with AN [5]. Sedham et al. postulated that hormonal imbalances in anorexia may contribute to poor smooth muscle development, leading to increased risk for arterial dissection. Vascular endothelial dysfunction is hypothesized to play a role in SCAD. A number of endocrine functions frequently altered in severe anorexia, support vascular endothelial health. Derangements in the hypothalamic-pituitary-thyroid axis often manifest in severe anorexia nervosa with low triiodothyronine (T3). T3 enhances endothelial and smooth muscle function and low T3 levels have a demonstrated association with cardiovascular disease [6]. However, thyroid function tests in this patient were confounded by a supratherapeutic levothyroxine dose. Significant changes in catecholamine metabolism have been observed in patients with AN when weight is 20% below ideal body weight [7]. Catecholamine surges have been previously implicated in the pathophysiology of SCAD. In fact, Chow et al. proposed that surges in catecholamines lead to vascular myointimal dysplasia and this may underlie the association observed between emotional stress and SCAD [8]. Additionally, estradiol levels are also often blunted in AN, and estradiol is protective of the vascular endothelium in several animal models [9].

A number of micronutrients often deficient in AN are important for vascular endothelial health. Zinc and selenium deficiencies are common in patients with severe anorexia [10] and have both been implicated as a mediator of inflammation in endothelial function [6, 11]. Vitamin C also has a well-established role in the development and maintenance of endothelial cells and the vascular basement membrane [12]. Recent studies have shown that patients with AN generally have increased inflammatory cytokines [13, 14]. This state of increased inflammation could lead to endothelial dysfunction, weakening the vascular intimal layer and predisposing to the development of SCAD. Finally, patients with AN may be at risk to mechanical injury to the vasculature. Prolonged retching, a behavior observed in patients with purging subtypes of eating disorders, has been identified as a precipitating factor in SCAD cases [15].

**Figure 1:**
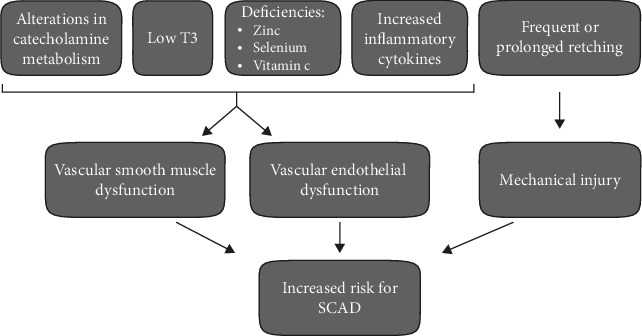
Hypothesized stressors predisposing to SCAD in patients with severe anorexia nervosa.

Figure 1 summarizes the proposed endocrine, nutritional, and physical stressors present in patients with severe anorexia that may predispose this population to SCAD. Considering our patient, it is possible that her preceding emotional and physical stress triggered her development of SCAD though she may have been predisposed to this injury due to her state of chronic severe malnutrition, endocrine dysregulation, and history of purging leading to a compromised vascular endothelium. This case adds to the one previously reported case of SCAD in AN. Further studies will be needed to understand whether patients with AN may be at increased risk for SCAD and may contribute to the high cardiac comorbidities in this population. Better understanding the relationship between AN and SCAD could inform treatment strategies aimed at the prevention of this potentially life-threatening cardiovascular complication.

## Data Availability

The underlying data supporting the content of the case report can be found in the Mass General Brigham Research Data Repository. Authors can make data available on request through the Mass General Brigham Institutional Review Board.
